# Long-Term Mobile-Based Glycemic Intervention for Secondary Prevention in Patients With Diabetes Undergoing Surgical Revascularization: Multicenter Randomized Controlled Trial

**DOI:** 10.2196/72226

**Published:** 2026-06-30

**Authors:** Yangwu Song, Yifeng Nan, Xieraili Tiemuerniyazi, Ziang Yang, Shicheng Zhang, Xi Li, Wei Feng

**Affiliations:** 1 Department of Cardiovascular Surgery, Fuwai Hospital, Chinese Academy of Medical Sciences and Peking Union Medical College, Fuwai Hospital, Beilishi Road No. 167, Xicheng District, Beijing, 100037, China, +86 101088322261; 2 National Clinical Research Center for Cardiovascular Diseases, State Key Laboratory of Cardiovascular Disease, Fuwai Hospital, National Center for Cardiovascular Diseases, Fuwai Hospital, Chinese Academy of Medical Sciences and Peking Union Medical College, Beijing, China

**Keywords:** cardiovascular disease, coronary artery disease, coronary artery bypass, diabetes mellitus, metabolic syndrome, mobile health, secondary prevention, glycemic control, glycohemoglobin, quality of life

## Abstract

**Background:**

Despite the growing amount of patients who underwent coronary artery bypass grafting (CABG) in low- and middle-income countries like China, their glucose control was suboptimal, likely due to poor adherence to healthy lifestyles and preventive medications. Mobile health tools facilitating secondary prevention seem promising, but evidence focusing on this high-risk population is scarce.

**Objective:**

This study aimed to evaluate the significance of mobile health tools in long-term glycemic management for post-CABG patients with comorbid diabetes mellitus.

**Methods:**

GUIDEME (glycemic control using mini program–based intervention in patients with diabetes undergoing coronary artery bypass to promote self-management) is a multicenter, open-label, closed-user group, randomized controlled trial, in which 1066 patients with diabetes who had recently undergone CABG were enrolled and allocated into 2 groups. Patients in the control group received conventional health education before discharge, whereas those in the intervention group additionally received automatic delivery of bite-sized health education and medication reminders through a smartphone app during the 6 months after discharge. The primary end point was a change in glycosylated hemoglobin (HbA_1c_) from baseline to 6 months.

**Results:**

Among the 1066 eligible participants enrolled, a total of 1038 (97.4%) had completed the follow-up, while 1000 (93.8%) had 6-month HbA_1c_ results available. Although only 79 (14.9%) patients in the intervention group were defined as active users, a greater reduction of HbA_1c_ in the intervention group was observed (adjusted between-group mean difference −0.13, 95% CI −0.25 to −0.01; *P*=.04). The intervention group also had a high proportion of good medication adherence (96.1% vs 93.2%, *P*=.04). There was no difference between the 2 groups regarding the secondary end points.

**Conclusions:**

Health education and medication reminders based on smartphone app achieved a statistically significant but modest between-group difference in HbA_1c_, the clinical relevance of which remains uncertain.

## Introduction

Coronary artery disease (CAD) is one of the leading causes of death globally [1]. It is noteworthy that in low- and middle-income countries, the prevalence and mortality of CAD have been growing dramatically [2]. Meanwhile, coronary artery bypass grafting (CABG) has been increasingly adopted as a beneficial therapy, accompanied by improvements in treatment capacity [3]. In China, for example, the overall volume of CABG has increased from 9802 in 2013 to 10,027 in 2018 [4]. However, substantial gaps exist in secondary prevention after CABG, which led to suboptimal prognosis. In particular, a larger proportion of diabetes mellitus (DM) patients in China had failed to achieve ideal long-term glucose control [5], which may be caused by poor medication adherence and unhealthy lifestyles [6]. As demonstrated by a recent study focusing on Chinese patients after CABG, the adherence rate to secondary prevention medications was merely 50% at the 6-month postoperative follow-up [7]. Furthermore, a multicenter observational study reported that nearly 75% of patients with diabetes in China failed to achieve optimal glycemic control. Poor glycemic control was significantly associated with suboptimal medication adherence (odds ratio [OR] 1.63, 95% CI 1.08‐2.46; *P*=.02) [8]. These findings highlight a substantial gap between guideline-recommended care and real-world practice in this high-risk population.

Mobile health (mHealth) leveraging mobile internet technologies is able to provide coherent support for health education and self-management to patients with chronic conditions [9]. The practical needs are greater for those facing geographic barriers [10], like in China, nearly one-fourth of the patients receiving CABG in 2021 were on interprovincial medical tours [11], which makes routine postdischarge care more difficult. However, the findings in prior studies about the effectiveness of mHealth tools on glycemic control were inconsistent [12-16], probably due to varied intervention approaches (mostly done through essays or short messages) [17-19] and different participants’ characteristics (sex, age, and educational background) [20,21]. Despite the growing popularity of short-form videos, which have a much lower literacy threshold and can use fragmented time, the application of video-based health education is still scarce.

To close this knowledge gap, we implemented the GUIDEME (glycemic control using mini program–based intervention in patients with diabetes undergoing coronary artery bypass to promote self-management) study, a multicenter, open-label, randomized controlled trial (RCT). The hypothesis of this study is that video-based health education and medication reminders through the smartphone app can improve glycemic control in patients with diabetes who underwent CABG.

## Methods

### Study Overview

GUIDEME was a parallel, 1:1, open-labeled, RCT. The detail of the GUIDEME trial was published elsewhere [22]. The design of this trial was registered at National Library of Medicine (NCT04192409) [23]. Participants were enrolled during January 2020 and October 2021, and the last follow-up was completed in June 2022. This study was reported in accordance with the CONSORT-EHEALTH (Consolidated Standards of Reporting Trials of Electronic and Mobile Health Applications and Online Telehealth) guidelines (see Checklist 1).

### Ethical Considerations

This trial was approved by the Institutional Review Board of Fuwai Hospital (2019‐1151) in June 2019. All participants provided written informed consent at the screening visit. Participants did not receive any financial compensation or incentives for participating in this study. All data were deidentified for protection of the patients’ privacy. The study was conducted in accordance with the principles of the Helsinki Declaration.

### Participants

Patients diagnosed with type 2 diabetes, who underwent isolated CABG, were deemed eligible. Patients were excluded if they (1) could not read the health educational materials, defined as a self-reported educational level below elementary school graduation or an inability to read Chinese characters fluently; (2) had cognitive or communication disorders; (3) had no ability to use or access to a smartphone, and (4) died before discharge.

Recruitment was conducted in 2 cardiac surgery centers simultaneously. In each center, patients who were hospitalized for CAD with DM and had undergone CABG were included. The diagnoses of CAD and DM, as well as the indications for surgical revascularization, were adjudicated centrally based on the patients’ medical records. Once potentially eligible individuals were identified, study staff explained the study protocol to them face-to-face and invited them to participate. A written informed consent form containing detailed study information was also provided to the patients. Baseline characteristics data were recorded after the enrollment. The sex of the participants was determined by biological factor only.

Type 2 diabetes mellitus was diagnosed according to the Standards of Medical Care in Diabetes–2019. Diagnosis was based on the presence of classic symptoms of hyperglycemia (eg, polyuria, polydipsia, polyphagia, or unexplained weight loss) plus a random plasma glucose ≥11.1 mmol/L, fasting plasma glucose ≥7.0 mmol/L, a 2-hour plasma glucose of ≥11.1 mmol/L during an oral glucose tolerance test, or glycosylated hemoglobin (HbA_1c_) level of ≥6.5%. All diagnoses were further reviewed and confirmed by the attending physician based on these criteria [24].

To ensure procedural consistency across the 2 centers, several measures were implemented prior to study initiation: (1) standardized operating procedures were established for patient recruitment, informed consent, baseline data collection, intervention delivery, and outcome assessment; (2) all participating staff, including nurses and follow-up coordinators, received centralized training using identical materials to ensure uniform implementation and follow-up procedures; and (3) key laboratory measurements, including HbA_1c_, were analyzed using the same model of equipment (ADAMS A1c HA-8180 [ARKRAY Inc]) in certified laboratories at each center to minimize intercenter variability.

### Allocation and Intervention

Participants were allocated to the intervention or control group in a 1:1 ratio using a centralized, computer-based minimization randomization system after enrollment. Minimization was performed based on age, sex, education level, medical insurance status, and history of myocardial infarction, with all factors assigned equal weight. To reduce predictability, a probabilistic element was incorporated, whereby 90% of allocations followed the minimization criterion and 10% were assigned at random. The first participant was allocated with equal probability to either group. The randomization sequence was generated and managed by an independent statistician and was concealed from investigators and outcome assessors throughout the trial.

Participants in the control group received conventional health education before discharge, while those in the intervention group additionally received videos and text messages for health education and medication reminders through a smartphone app based on WeChat for 6 months after discharge. The intervention tool was a custom-built app specifically for this study, which uses a closed registration system. Upon obtaining consent and completing randomization to the intervention arm, an account was registered for the patient by study personnel during a face-to-face session, which also included training on app use.

In the intervention app, the educational materials (a cluster of 180 videos) were developed by a multidisciplinary team of cardiac surgeons, cardiologists, endocrinologists, psychologists, nurses, and public health researchers based on clinical guidelines [25-29]. The themes of the health education included (1) general knowledge about CAD and DM, (2) postoperative antiplatelet agents, (3) lipid-lowering therapy, (4) beta-blockers, (5) glucose monitoring and control, (6) blood pressure control, (7) smoking cessation, (8) cardiac rehabilitation, and (9) lifestyle recommendations such as weight loss, physical activity, and diet. The team used behavioral change techniques (BCTs) to develop short-form videos by setting goals, providing information on the consequences of behavior, providing feedback on performance, social support, and so on [30]. All videos were shorter than 2 minutes. The educational information was automatically pushed to patients in a preset order according to the time of enrollment. The frequency of delivery was set to once a day during 6 months.

In addition, participants in the intervention group could receive reminders about taking secondary prevention medications, including antiplatelet agents, beta-blockers, nitrates, lipid-lowering agents, oral hypoglycemic agents, and insulin. For each participant, the prescribed medications were entered at enrollment and can be modified afterwards.

### Outcomes and Ascertainment

The primary outcome was the change in HbA_1c_ between the baseline and the 6-month follow-up. Secondary outcomes included changes in the proportion of patients achieving an HbA_1c_ less than 7%, systolic blood pressure (SBP), low-density lipoprotein cholesterol (LDL-C), fasting blood glucose (FBG), and major adverse cerebrovascular and cardiovascular events (MACCEs), which were defined as a composite of all-cause death, nonfatal myocardial infarction, stroke, repeat revascularization (defined as any subsequent coronary revascularization procedure, including percutaneous coronary intervention or repeat CABG, involving either target or nontarget vessels), and cardiac rehospitalization.

Self-reported medication adherence and quality of life (QoL) questionnaire were collected only at 6 months and analyzed as exploratory outcomes. User data of patients who were allocated to the intervention group were extracted at 6 months. Based on prior studies indicating that sustained engagement with mHealth interventions is typically observed in approximately 10% of users [31], participants in the top decile of app usage frequency were defined as “active users.” In this study, this corresponded to a threshold of 12 app launches or more, which also approximated engagement during at least half of the 24-week intervention period.

The follow-up was conducted every 3 months after enrollment, which was completed by telephone or in-person visit. Outcomes were collected and assessed at 6-month follow-up. HbA_1c_, LDL-C, FBG, and SBP measured at outpatient clinics of the study sites were preferred. In study sites, HbA_1c_ was determined using a high-performance liquid chromatography technique with the same model of analyzer (ADAMS A1cHA-8180 [ARKRAY Inc.]) to ensure methodological consistency.

In the follow-up visits, if MACCEs were reported by the participants or their families, central adjudication based on the medical records was conducted. The trial was conducted in an open-label manner; however, laboratory personnel and staff responsible for follow-up outcome assessment were blinded to group allocation to minimize potential assessment bias. In the meantime, medication adherence was collected by a self-reported questionnaire. The QoL was measured using the short version of EQ-5D and EuroQol-Visual Analogue Scale. The questionnaire was administered either online or in person, according to the patient’s preference.

### Statistical Analysis

A sample size of 820 was estimated to provide 80% power at the significance level of .05 to detect a 0.3% absolute difference in HbA_1c_ change, assuming a mean HbA_1c_ level of 7% (SD 1.4%) at baseline based on data from studies involving similar populations [19]. This sample size allowed for a 20% loss to follow-up during the study period. During the course of the study, the COVID-19 pandemic led to restrictions on interregional travel and reduced accessibility to follow-up visits, raising concerns about a higher-than-expected dropout rate. To mitigate the potential impact of these unforeseen circumstances and to preserve statistical power, the recruitment target was increased, resulting in the enrollment of 1066 participants.

Baseline patient characteristics are presented as means with SDs for continuous variables that are normally distributed or as median with IQR. Categorical variables are presented as numbers with percentages. Baseline comparisons for continuous variables between groups are performed using Student *t* test or a nonparametric test as appropriate. Categorical variables were analyzed using the chi-square test or Fisher exact test.

Available-case analysis was conducted for comparisons of primary and secondary outcomes. Additionally, for the missing follow-up data, a sensitivity analysis was performed based on the intention-to-treat principle using baseline observation carried forward for the imputation of missing data. Between-group comparisons for the continuous outcomes including the primary end point (change in HbA_1c_ at 6 mo) were performed using an analysis of covariance (ANCOVA) model adjusting for baseline HbA_1c_. This analytic approach was prespecified in the study protocol. Results were reported as adjusted mean differences with corresponding 95% CIs and exact *P* values. Log-binomial regression was used to compare the change in proportions of patients achieving HbA_1c_≤7% between groups. The QoL questionnaire, including EQ-5D and EuroQol-Visual Analogue Scale, was compared by nonparametric test. Self-reported medication adherence was analyzed as a categorical outcome by chi-square test. Log-rank test was used to compare the occurrence of MACCEs over time.

Additionally, we performed prespecified subgroup analyses of primary outcomes by baseline age (<60 and ≥60 y), sex (male and female), area (urban and rural), educational level (≤12 and >12 y), and tertile of baseline HbA_1c_, using ANCOVA.

To explore the factors associated with adherence to the intervention, logistic regression was conducted to identify the characteristics of frequent users in the intervention group. Variables with *P*<.10 in univariate regression were included in the multivariate regression.

All tests of significance were 2-tailed, with α set at .05. Analyses were performed using SPSS software (version 26.0.0.0; IBM Corp).

## Results

### Baseline and Perioperative Characteristic

Between January 1, 2020, and September 29, 2021, 1066 eligible participants were enrolled and assigned to the intervention (n=534) or control group (n=532). Recruitment was ended after the designated sample size was reached. Among the 1038 patients who completed the final follow-up, 1000 provided primary end point data, and our primary analysis was based on this population of 1000 participants.

In this overall cohort, the mean age was 60.9 (SD 8.2) years, and 210 out of 1038 (20.2%) were female; 338 out of 1038 (32.6%) patients underwent myocardial infarction. In total, 909 out of 1038 (87.6%) patients were educated below master's degree. The urban medical insurance was the most commonly used (420/1038, 40.5%). The baseline characteristics of participants were comparable between the 2 groups (Table 1). No significant differences were observed regarding perioperative characteristics or discharge prescriptions (Table S1 in Multimedia Appendix 1).

**Table 1.** Baseline clinical and social characteristics of patients included in the final analysis.

Variables	Control group (n=486)	Intervention group (n=514)
Female, n (%)	99 (20.4)	104 (20.2)
Age (y), mean (SD)	60.8 (8.1)	60.9 (8.3)
BMI (kg/m^2^), median (IQR)	25.8 (23.8-27.7)	25.6 (23.9-27.7)
SBP^a^ (mm Hg), median (IQR)	134 (122-148)	133 (122-144)
Cigarette and alcohol, n (%)	219 (45.1)	245 (47.7)
Hypertension, n (%)	338 (69.5)	356 (69.3)
Hyperlipidemia, n (%)	377 (77.6)	399 (77.6)
Prior stroke, n (%)	51 (10.5)	44 (8.6)
NYHA^b^ class III or IV, n (%)	128 (26.3)	131 (25.5)
Triple-vessel disease, n (%)	449 (92.4)	472 (91.8)
LM^c^ disease, n (%)	127 (26.1)	133 (25.9)
Preoperative medication, n (%)		
Beta blocker	441 (90.7)	451 (87.7)
ACEI^d^ or ARB^e^	180 (37.0)	182 (35.4)
Statin	449 (92.4)	480 (93.4)
Nonstatin hypolipidemia agents	86 (17.7)	78 (15.2)
Hypoglycemic intervention, n (%)		
Oral medication	245 (50.4)	276 (53.7)
Insulin	206 (42.4)	192 (37.4)
None	35 (7.2)	46 (8.9)
Diabetic comorbidity, n (%)	16 (3.3)	18 (3.5)
Preoperative laboratory		
HbA_1c_^f^ (%), median (IQR)	7.5 (6.7-8.5)	7.5 (6.8-8.4)
HbA_1c_≤7.0%, n (%)	177 (36.4)	178 (34.6)
Creatine (mmol/L), median (IQR)	83.7 (72.3-95.0)	82.4 (73.4-92.8)
FBG^g^ (mmol/L), median (IQR)	7.1 (5.7-8.9)	6.9 (6.0-8.4)
LDL-C^h^ (mmol/L), median (IQR)	2 (1.6-2.5)	2.0 (1.6-2.5)
Current residence, n (%)		
City	326 (67.1)	326 (63.4)
Rural area	160 (32.9)	188 (36.6)
Enrollment center, n (%)		
Fuwai Hospital	459 (94.4)	476 (92.6)
Qingdao Fuwai Hospital	27 (5.6)	38 (7.4)
Health insurance, n (%)		
Urban basic health insurance	197 (40.5)	206 (40.1)
Rural health insurance	117 (24.1)	127 (24.7)
Other health insurance	172 (35.4)	181 (35.2)
Education level, n (%)		
Below senior high school	216 (44.4)	234 (45.5)
Senior high school or specialty	209 (43.0)	218 (42.4)
Bachelor’s degree or above	61 (12.6)	62 (12.1)

a SBP: systolic blood pressure.

b NYHA: New York Heart Association.

c LM: left main.

d ACEI: angiotensin-converting enzyme inhibitors.

e ARB: angiotensin receptor blocker.

f HbA_1c_: glycosylated hemoglobin.

g FBG: fasting blood glucose.

h LDL-C: low-density lipoprotein cholesterol.

### Follow-Up

**Figure 1.**
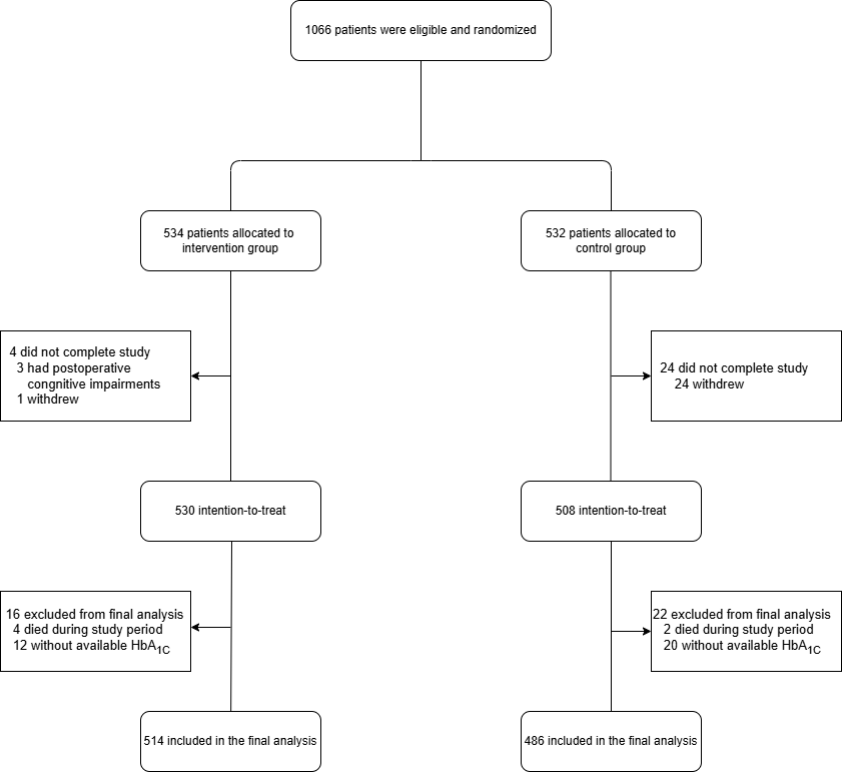
Flowchart of patients’ recruitment. HbA_1c_: glycosylated hemoglobin.

After group allocation, 3 participants were unable to engage with the intervention due to postoperative complications, and 25 patients withdrew during follow-up. A comparison of baseline characteristics between participants who completed the study and those who withdrew was conducted, and the results showed that patients who withdrew had higher LDL-C levels (2.0 [1.6, 2.5] vs 2.3 [1.9, 2.8] mmol/L, *P*=.03), while other baseline characteristics are similar (Table S2 in Multimedia Appendix 1). Considering that the majority of dropouts occurred in the intervention group, we conducted a further analysis comparing the baseline characteristics of completers versus dropouts within this group. The results showed no significant difference (Table S3 in Multimedia Appendix 1). Finally, a total of 1038 patients completed the study (530 in intervention group vs 508 in control group) (Figure 1). Among them, 6 (0.6%) participants died during the follow-up (4 in the intervention group vs 2 in the control group). In the cohort, 1000 (96.4%) patients had available 6-month HbA_1c_ results and were included in primary and secondary analyses. Participants excluded from the primary analysis had higher baseline creatinine (median 92.1, IQR 76.3-106.0 mmol/L vs 83.0, IQR 72.9-94.0 mmol/L; *P*=.04) but did not differ in other major characteristics (Table S4 in Multimedia Appendix 1).

### Outcome Assessment

The unadjusted within-group reductions in HbA_1c_ were −0.70% in the intervention group and −0.50% in the control group. A greater decrease from baseline to 6-month HbA_1c_ was attained in the intervention group; the adjusted between-group mean difference derived from the ANCOVA model was −0.13% (95% CI −0.25 to −0.01; *P*=.04; Tables2  3). Given that an HbA_1c_ reduction of ≥0.5% is often regarded as clinically meaningful [27], we performed a post hoc exploratory analysis to assess this outcome. After adjustment for baseline HbA_1c_, a greater proportion of patients in the intervention group achieved a clinically meaningful HbA_1c_ reduction of ≥0.5% compared with the control group (relative risk 1.14, 95% CI 1.01‐1.28; *P*=.03; Table S6 in Multimedia Appendix 1). The finding of the primary outcome remains consistent in the intention-to-treat sensitivity analysis using baseline observation carried forward; the between-group adjusted mean difference remained statistically significant (−0.14%, 95% CI −0.25 to −0.02; *P*=.03; Table S5 in Multimedia Appendix 1). The number of patients with HbA_1c_ level of no more than 7% increased from 178 (34.6%) to 295 (57.4%) in the intervention group and from 177 (36.4%) to 256 (52.7%) in the control group. There was no significant difference between groups (relative risk 0.93, 95% CI 0.84‐1.03; *P*=.18; Tables2  3). The absolute risk difference was 4.7% (95% CI −1.6 to −11.0%), with a corresponding number needed to treat of 21.2, suggesting a trend favoring the intervention. Comparisons in secondary outcomes including SBP, FBG, and LDL-C showed no significant differences (Table 3).

**Table 2.** Primary and secondary end point at baseline, follow-up, and change from baseline.

Variables	Control group		Intervention group		Adjusted *P* value
	Follow-up	Change	Follow-up	Change	
HbA_1c_^a^ (%), median (IQR)	7.0 (6.5 to 7.7)	−0.3 (−1.3 to 0.4)	6.9 (6.4 to 7.5)	−0.6 (−1.5 to 0.2)	.04
HbA_1c_ ≤7.0%, n (%)	256 (52.7)	—^b^	295 (57.4)	—	.18
SBP^c^ (mmol/L), median (IQR)	130 (120 to 136)	−6 (−19 to 6)	130 (120 to 135)	−5 (−16 to 8)	.54
FBG^d^ (mmol/L), median (IQR)	7.0 (6.1 to 8.0)	0.1 (−1.5 to 1.4)	6.9 (6.1 to 7.8)	0 (−1.5 to 1.3)	.35
LDL-C^e^ (mmol/L), median (IQR)	2.2 (1.7 to 2.8)	0.2 (−0.4 to 0.8)	2.2 (1.8 to 2.8)	0.2 (−0.4 to 0.8)	.46

a HbA_1c_: glycated hemoglobin.

b Not available.

c SBP: systolic blood pressure.

d FBG: fasting blood glucose.

e LDL-C: low-density lipoprotein cholesterol.

**Table 3.** Mean difference in change of primary and secondary outcomes.

Variables	Mean difference in change, median (IQR)	Adjusted mean difference in change, median (IQR)	Adjusted *P* value
HbA_1c_^a^ (%)	−0.1 (−0.2 to 0)	−0.1 (−0.3 to −0.01)	.04
HbA_1c_ ≤7.0%	—^b^	0.9 (0.8 to 1.0)^c^	.18
SBP^d^ (mmol/L)	−0.7 (−2.2 to 0.8)	−0.5 (−2.0 to 1.0)	.54
FBG^e^ (mmol/L)	−0.2 (−0.4 to 0.1)	−0.1 (−0.4 to 0.1)	.35
LDL-C^f^ (mmol/L)	0 (−0.1 to 0.1)	0 (−0.6 to 0.1)	.46

a HbA_1c_: glycated hemoglobin.

b Not available.

c Reported as relative risk (95% CI).

d SBP: systolic blood pressure.

e FBG: fasting blood glucose.

f LDL-C: low-density lipoprotein cholesterol.

Compared with the control group, participants in the intervention group were more likely to report “hardly forget to take their medicine” (96.1% vs 93.2%; *P*=.047). The quality of life was comparable between the 2 groups. MACCE occurred in 22 individuals during the follow-up (12 in the intervention group vs 10 in the control group); the overall MACCE incidence was 4.29 per 100 person-years (3.98 in the control group vs 4.59 in the intervention group), with no difference observed between the groups (hazard ratio 1.15, 95% CI 0.50‐2.67; *P*=.74; Table S6 in Multimedia Appendix 1).

### Factors Related to the Effectiveness and Adherence of Intervention

**Figure 2.**
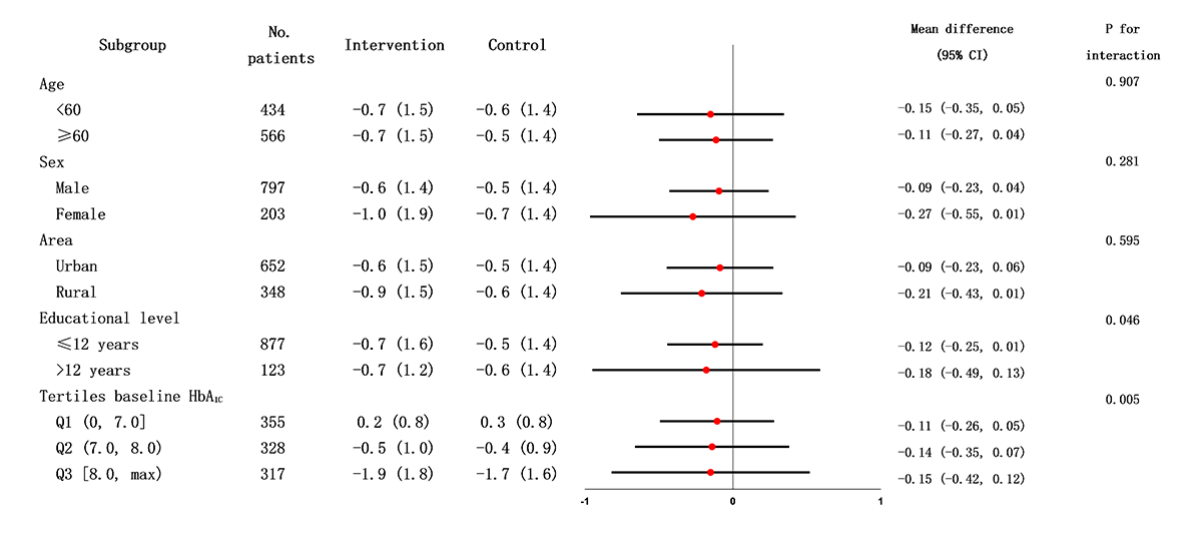
Subgroup analyses of mobile intervention. HbA_1c_: glycosylated hemoglobin.

There was no significant interaction between the intervention and the prespecified subgroups except for baseline tertiary HbA_1c_ and educational level ≤12 versus >12 years. None of the subgroups showed a significantly greater change in HbA_1c_ levels in the intervention group compared to the control group (Figure 2).

App usage data were extracted from 530 patients in the intervention group. The median total launch count was 5.5 (IQR 3.0-9.3) times, and 79 (14.9%) patients were active users of the app. Patients with a history of cigarette and alcohol use (OR 0.50, 95% CI 0.28-0.88; *P*=.02), with baseline HbA_1c_ ≤7% (OR 0.54, 95% CI 0.31-0.96; *P*=.04), and who lived in urban areas (OR 0.51, 95% CI 0.31-0.83; *P*=.01) were less likely to become active users of the intervention (Table S7 in Multimedia Appendix 1). The model fit was acceptable (likelihood ratio *χ*²(5)=26.327, *P*<.001, and the Hosmer-Lemeshow test *χ*²(8)=5.440, *P*=.61), and no evidence of multicollinearity was observed among the variables included in the multivariable model (all variance inflation factor <5.0; Table S7 in Multimedia Appendix 1).

**Figure 3.**
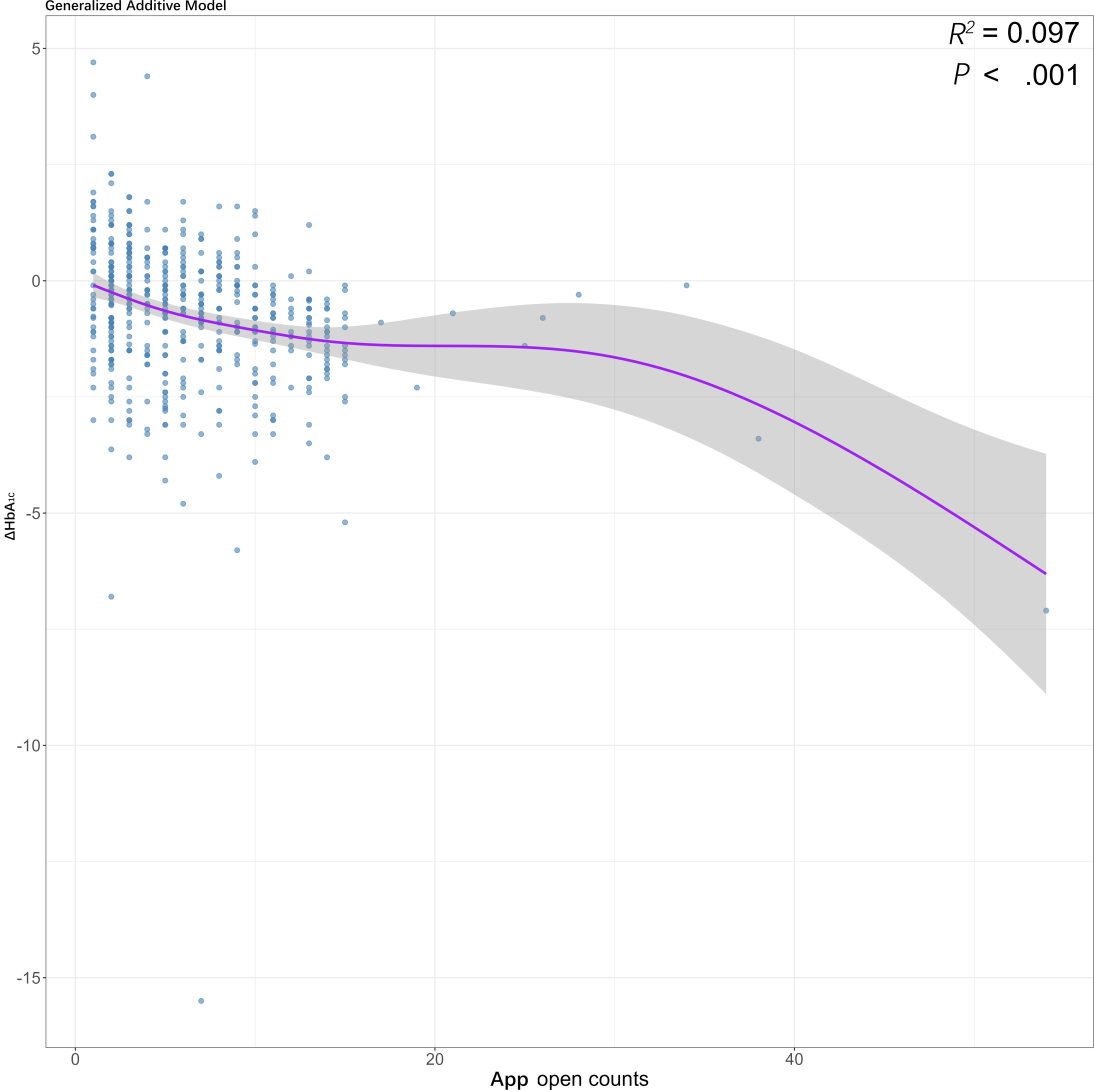
Dose-response analysis of the relationship between app adherence and the primary outcome. GAM: generalized additive model; HbA_1c_: glycosylated hemoglobin.

Among participants in the intervention group, a significant association was observed between app usage frequency and the magnitude of HbA_1c_ reduction. Participants with higher numbers of app launches tended to exhibit greater decreases in HbA_1c_ (regression coefficient −0.09, 95% CI −0.11 to −0.06; *P*<.001). On average, an increase of 10 app launches was associated with an estimated −0.86% (95% CI −1.10 to −0.62) change in HbA_1c_. This pattern is consistent with a dose-response–like association; however, given the observational nature of this analysis, the findings should be interpreted as associative rather than causal (Figure 3).

Subgroup analyses of app user data indicated heterogeneity in usage patterns. Participants in the lowest baseline HbA_1c_ tertile (Q1) demonstrated fewer app launches and a lower proportion of active users. Higher app engagement was more frequently observed among female participants and those residing in rural areas (Table S7 in Multimedia Appendix 1). Across most subgroups, participants classified as active app users experienced greater reductions in HbA_1c_ than nonactive users. Exceptions were observed in participants with college-level education or above and in those within the highest baseline HbA_1c_ tertile (Q3), in whom HbA_1c_ reductions were comparable between active and nonactive users (Table S8 in Multimedia Appendix 1).

## Discussion

### Principal Findings

In this RCT among high-risk individuals who underwent CABG, short-form videos and text messages based on a smartphone app for health education and medication reminders seem to improve glycemic control. The reduction in HbA_1c_ was statistically greater in the intervention group; however, the magnitude of the between-group difference was modest. Although only 14.9% of patients in the intervention group remained active app users by the end of the study, the effectiveness of the intervention is positively correlated with the strength of app usage.

### Comparison to Prior Work

The novelty of our study lies in its specific focus on the high-risk population of patients with diabetes undergoing CABG and its adoption of a video-centric approach for information delivery. Patients with diabetes undergoing CABG represent a particularly high-risk population due to the convergence of multiple clinical and physiological risk factors. Previous studies have consistently shown that diabetes and poor glycemic control are associated with increased mortality and a higher incidence of postoperative complications following CABG [32,33]. In the perioperative and early postoperative period, surgical stress, pain, inflammatory responses, and dietary restrictions contribute to stress-induced hyperglycemia through alterations in counter-regulatory hormones such as cortisol, catecholamines, and glucagon, thereby increasing glycemic variability and complicating glucose management [34]. Moreover, post-CABG patients frequently present with multiple cardiometabolic comorbidities and require complex medication regimens, which further challenge sustained glycemic control and long-term adherence.

Consistent with these mechanisms, prior studies have demonstrated that elevated postoperative blood glucose levels are associated with increased risks of myocardial infarction, repeat revascularization, and long-term cardiovascular events [35,36]. Accordingly, major clinical guidelines recommend maintaining HbA_1c_ at ≤7% in this high-risk population as part of comprehensive secondary prevention strategies [25]. Although current guidelines for coronary revascularization do not explicitly recommend mHealth as an intervention, the importance of achieving optimal glycemic control as part of secondary prevention in cardiac rehabilitation for patients with coronary heart disease has been emphasized by the clinical guidelines [25,37]. Moreover, a meta-analysis by Jin et al supports the significant role of mHealth in enhancing secondary prevention outcomes in coronary heart disease [38].

While mHealth has demonstrated potential benefits in the management of type 2 diabetes, most existing interventions are not readily applicable to diabetic patients following CABG surgery. This limited applicability largely reflects the distinct clinical complexities of this population. First, post-CABG patients are typically required to manage multiple concurrent medications, including glucose-lowering agents, antiplatelet therapies, statins, and other cardiovascular drugs, which increases the risk of nonadherence and potential drug-drug interactions. Generic diabetes-focused mHealth apps rarely incorporate coordinated reminders or integrated management strategies for such complex medication regimens. Second, postoperative recovery after CABG extends beyond glycemic control to include wound care, pain management, graded physical rehabilitation, and surveillance for cardiovascular complications such as angina or heart failure. Effective secondary prevention in this context therefore requires an intervention capable of simultaneously addressing glycemic control and cardiovascular risk management, rather than focusing on glucose targets alone. Consistent with this, prior studies have shown that patients with diabetes after CABG often exhibit suboptimal achievement of multiple secondary prevention targets, including glucose, lipid, and blood pressure control [39].

We selected video as the core modality for delivering educational content based on several considerations. Patients with diabetes undergoing CABG are often older and may experience difficulties with text-based educational materials; in this context, video presentations with subtitles may offer greater accessibility. Previous mHealth interventions targeting post-CABG or similar high-risk populations have reported limited effectiveness when relying primarily on text-based approaches [31]. Although direct comparative evidence between video- and text-based interventions in this specific population is currently lacking, studies in other chronic disease populations suggest that supplementing text with video components may enhance engagement and intervention effectiveness [40]. By adapting the intervention format accordingly, we aimed to improve the acceptability and potential effectiveness of mHealth interventions for patients with diabetes following CABG.

### Strengths and Limitations

As the first RCT on mHealth tools facilitating glucose control in high-risk patients who underwent CABG, this study extended the literature in several ways. First, the intervention group demonstrated a greater within-group reduction in HbA_1c_ (0.7%). This is consistent with a meta-analysis on mHealth intervention, which reported a mean reduction of 0.42% [41]. Although the relative increase in HbA_1c_ target attainment did not reach statistical significance, the absolute risk difference of 4.7% corresponds to a number needed to treat of approximately 21, suggesting a modest but potentially clinically relevant absolute benefit at the population level. Although a greater proportion of patients in the intervention group achieved a clinically meaningful reduction in HbA_1c_, this finding arose from a post hoc exploratory analysis and should not be considered confirmatory; instead, it provides only tentative support that the intervention may have operated in the expected direction. It is also noteworthy that in this study, HbA_1c_ in the control group also decreased, probably because the surgery itself could act as a trigger for changing the attitude and behavior of patients. The effect has basically met the expectation—according to current guidelines, an intervention is considered effective when it leads to an absolute reduction of 0.3% or more [42]. Furthermore, the reduction in HbA_1c_ of 0.7% is estimated to reduce the risk of diabetes-related mortality by 14.7% based on available evidence [43]. However, the adjusted between-group effect size was modest (−0.13%) and below the prespecified clinically meaningful threshold of 0.3%. Therefore, the clinical significance of the observed improvement remains uncertain. It should also be noted that the number of MACCEs observed during follow-up was relatively small, which limited the statistical power to detect between-group differences; therefore, the absence of a statistically significant effect on MACCEs should not be interpreted as evidence of no effect.

The observed improvement in HbA_1c_ is likely attributable to the structured integration of multiple BCTs embedded in the intervention, rather than simple information delivery alone [44,45]. Specifically, techniques such as goal setting and planning, self-monitoring with feedback, and knowledge shaping may have enhanced patients’ self-regulatory capacity for glycemic management, while automated medication reminders directly targeted medication self-management—a key determinant of glycemic control in this population. Together, these complementary BCTs provide a plausible behavioral mechanism underlying the observed glycemic benefit.

The greater improvement in glycemic control in the intervention group might be caused by better medication adherence. Undoubtedly, medication adherence is deemed one of the key links—but also the weak link—in secondary prevention, which is particularly true for glycemic management [46]. The prescription of secondary prevention medications increased significantly in patients with established CAD in China during the past 2 decades [47]. However, for long-term management, compliance would fall over time after discharge [48]. In particular, patients who underwent CABG were found to be generally less likely to use secondary preventive medications consistently after the procedure compared with patients who received percutaneous coronary intervention [49]. Like in our trial, prior studies have also found similar results that mHealth interventions can improve medication adherence [50-55]. Most of the interventions in these studies were medication reminders, and some of them involved dedicated medication reminder devices or constant instruction from medical personnel [52,55]. The results of our study highlighted the importance of mHealth intervention focusing on medication adherence and suggested that multiple functions on that should be considered.

Notably, subgroup analysis revealed a significant interaction with baseline HbA_1c_ tertiles, indicating that patients with higher baseline HbA_1c_ derived greater benefit from the intervention. This finding is clinically intuitive, as patients with poor baseline glycemic control are more likely to exhibit pronounced deficits in self-management behaviors, leaving greater room for improvement. In contrast, patients with relatively well-controlled baseline HbA_1c_ may already have established management routines, limiting the incremental benefit of additional interventions. This interaction suggests that mHealth-based interventions may be particularly valuable when targeted toward patients with suboptimal baseline glycemic control.

We also observed that higher levels of engagement with the intervention app were associated with greater reductions in HbA_1c_. In exploratory analyses restricted to participants in the intervention group, more frequent app use corresponded to larger improvements in glycemic control, yielding a dose-response–like pattern. Similar results were observed in a previous study [14]. However, this post hoc analysis was observational and may be influenced by residual confounding, self-selection, and reverse causality. Accordingly, these findings should be interpreted as hypothesis-generating rather than confirmatory and warrant validation in studies specifically designed to evaluate engagement-driven effects.

Previous studies have shown that it is challenging to maintain a high adherence to mHealth intervention. Evidence from reviews of chronic disease and cardiovascular health apps indicates that long-term active use rates barely reach 16.6% [56]. A prior trial (MISSION-2) reported that the use rate of the intervention app had dropped to less than 10% after 6 months [31], while its researchers believed that the poor compliance with the intervention app was one of the main reasons why their intervention had reached negative results. In GUIDEME, we had chosen short-form video, a rapidly expanding media type on mobile internet, as the major format of our education materials in the hope of achieving better compliance. Nevertheless, despite exploratory efforts to enhance engagement through short-form video delivery, overall user adherence in our study remained suboptimal, which may have attenuated the observed intervention effect and limited the internal validity and generalizability of the findings. Therefore, how to further enhance patients’ interest and compliance with mHealth intervention and alter their behavior in receiving health education information will be a critical concern determining the value of similar interventions and need further exploration in the future. An interesting finding is that urban residents were less likely to become active app users, possibly due to easier access to in-person care, which reduces reliance on digital tools. In contrast, rural patients may adopt remote interventions more readily. This aligns with diffusion-of-innovation theory and suggests that implementation strategies should consider local health care contexts rather than assuming higher acceptance in urban areas. Taken together, these findings suggest that the app-based intervention should be viewed as a low-intensity, scalable support to routine post-CABG care, with a modest but measurable effect on glycemic control, rather than as a stand-alone therapeutic approach.

In contrast to the improvement observed in HbA_1c_, no statistically significant between-group differences were detected for secondary outcomes, including SBP, FBG, and LDL-C. In addition to limitations related to follow-up duration and statistical power, these findings may also reflect differences in the relative emphasis of intervention components. Specifically, the app was primarily designed to support glycemic management and medication adherence, whereas fewer than 10% of the educational videos (16/180) directly addressed blood pressure or lipid management, and these focused on general disease knowledge rather than intensive or personalized management strategies. This content distribution may partly explain the absence of significant between-group differences in blood pressure and lipid outcomes. Similar patterns have been reported in previous mHealth studies targeting cardiometabolic risk factors [57]. Future studies may help clarify whether interventions with more targeted or intensive components could yield broader effects across multiple cardiometabolic outcomes.

### Future Directions

The current study provided a feasible option to improve secondary prevention and relieve the public health burden of chronic conditions. The core function of our intervention was based on an automatic BCTs-based health education delivery system, mostly using short-form video to minimize threshold related to education levels. The system requires only an affordable initial investment to establish a video library for the target population and a scheduled delivery order. The operation of the system does not depend on massive medical manpower and resource investment. On the other hand, the smartphone-based system is scalable, which can effectively overcome the geographic and economic barriers and help numerous patients who have difficulties in accessing medical centers for long-term self-management at reasonable costs. We selected the app platform of WeChat (with over 1.2 billion monthly active users) rather than developing an independent app, which also suggests a feasible strategy to facilitate the popularizing of intervention tools.

### Limitations

Several limitations should be acknowledged. First, limitations in participant flow documentation may affect the assessment of internal validity. Information from the screening phase was not systematically collected, and specific reasons for participant withdrawal or missing outcome data were not consistently recorded. This restricts a comprehensive evaluation of potential selection bias and attrition bias. Second, the subgroup and interaction analyses were exploratory in nature. Sample sizes within certain strata were relatively small, which limits statistical power. Consequently, these findings should be interpreted as hypothesis-generating rather than confirmatory. Third, characteristics of the digital intervention itself may limit generalizability. Health education was delivered primarily through short-form videos. While this format enhances engagement and information density, it requires smartphones with sufficient performance and stable internet access. Such requirements may constrain feasibility in resource-limited or low-connectivity settings. Fourth, user-related digital barriers should be considered. Older individuals and patients unable to operate smartphones were excluded from participation, introducing potential selection bias and limiting applicability to populations with lower digital literacy. Fifth, medication adherence was assessed using self-reported measures rather than validated adherence scales or objective indicators. Self-reported adherence is subject to recall and social desirability bias and therefore provides limited measurement precision. Accordingly, adherence findings should be regarded as indicative and exploratory and are insufficient to support definitive causal inferences. Sixth, the definition of “active user” (≥12 app launches) was determined post hoc based on the observed distribution of app usage within this study and therefore involves a degree of subjectivity. Although this operational threshold was intended to identify participants with relatively higher engagement, it may not fully capture the multidimensional nature of adherence to digital interventions. The absence of standardized metrics for mHealth engagement limits comparability with other studies, and future research should adopt validated and harmonized measures of digital intervention adherence. Seventh, safety reporting focused on surgery- and disease-related clinical events. App-specific adverse effects—such as anxiety, privacy concerns, or data security issues—were not systematically assessed. Although health-related quality of life was measured using the EQ-5D, the absence of digital intervention-specific harm assessment may have led to underestimation of potential app-related adverse effects. Finally, the follow-up period was relatively short. As a result, the durability of the observed effects and the long-term impact of the mHealth intervention on clinical outcomes and engagement remain uncertain. Future studies with extended follow-up and iterative optimization of app functionalities are needed to address these limitations.

### Conclusion

Although compared with conventional care, mHealth intervention featuring short-form video health education and medication reminders achieved greater HbA_1c_ decrease after CABG, the effect size was modest and did not reach commonly cited thresholds for clinical relevance; therefore, the clinical significance of this improvement should be interpreted with caution. This mobile-based intervention may serve as a feasible adjunct to conventional secondary prevention in patients with diabetes undergoing CABG. Further research on assessing the long-term effectiveness, testing personalized information push, and improving the app stickiness is needed.

## Supplementary material

10.2196/72226 Multimedia Appendix 1 The results of sensitivity analyses, exploratory analyses, and subgroup analyses supporting the main findings of the study.

10.2196/72226 Checklist 1 CONSORT-EHEALTH checklist.

## Abbreviations

ANCOVA: analysis of covariance
BCT: behavioral change technique
CABG: coronary artery bypass grafting
CAD: coronary artery disease
CONSORT-EHEALTH: Consolidated Standards of Reporting Trials of Electronic and Mobile Health Applications and Online Telehealth
DM: diabetes mellitus
FBG: fasting blood glucose
GUIDEME: glycemic control using mini program-based intervention in patients with diabetes undergoing coronary artery bypass to promote self-management
HbA_1c_: glycosylated hemoglobin
LDL-C: low-density lipoprotein cholesterol
MACCE: major adverse cerebrovascular and cardiovascular event
mHealth: mobile health
OR: odds ratio
QoL: quality of life
RCT: randomized controlled trial
SBP: systolic blood pressure
