# Supplementary material for: Long-Term Mobile-Based Glycemic Intervention for Secondary Prevention in Patients With Diabetes Undergoing Surgical Revascularization: Multicenter Randomized Controlled Trial
Source: J Med Internet Res. 2026 Jun 30;28:e72226. doi: 10.2196/72226 (PMC13318208; doi:10.2196/72226)
Supplement: Multimedia Appendix 1 [file jmir-v28-e72226-s001.docx]

**Supplementary tables**

**Table S1. Perioperative and follow-up characteristic**

| **Variables** | **Control group** | **Intervention group** | **P-value** |
| --- | --- | --- | --- |
|  | **n = 508** | **n = 530** |  |
| Cardiopulmonary bypass, no (%) | 279 (54.9) | 282 (53.2) | .58 |
| CPB time (min), median [Q1, Q3] | 108.0 [87.0, 131.0] | 109.0 [85.0, 132.0] | .97 |
| Cross-clamping time (min), median [Q1, Q3] | 76.0 [62.0, 97.0] | 78.0 [61.0, 99.0] | .92 |
| Number of distal anastomosis (no), median [Q1, Q3] | 3 [3, 4] | 3 [3, 4] | .67 |
| Intubation time (hours), median [Q1, Q3] | 16.0 [12.0, 18.5] | 16.0 [12.0, 19.0] | .65 |
| ICU-stay (hours), median [Q1, Q3] | 45.0 [22.0, 87.0] | 45.0 [22.0, 88.0] | .50 |
| Postoperative hospital-stay (days), median [Q1, Q3] | 7.0 [7.0, 9.0] | 7.0 [7.0, 9.0] | .41 |
| Total hospital costs (10k yuan), median [Q1, Q3] | 11.45 [10.29, 13.01] | 11.50 [10.34, 12.86] | .86 |
| Postoperative laboratory |  |  |  |
| Creatine (mmol/L), median [Q1, Q3] | 83.0 [71.4, 101.1] | 85.8 [72.9, 98.6] | .45 |
| FBG (mmol/L), median [Q1, Q3] | 9.25 [7.8, 10.9] | 9.1 [7.7, 10.9] | .32 |
| Operative complication |  |  |  |
| Reoperation, no (%) | 8 (1.6) | 9 (1.7) | .88 |
| Stroke, no (%) | 3 (0.6) | 3 (0.6) | >0.999 |
| Discharge medication |  |  |  |
| Aspirin, no (%) | 496 (97.6) | 518 (97.7) | .92 |
| Clopidogrel, no (%) | 405 (79.7) | 434 (81.9) | .38 |
| Beta blocker, no (%) | 460 (90.6) | 467 (88.1) | .20 |
| ACEI/ARB, no (%) | 31 (6.1) | 47 (8.9) | .09 |
| Statin, no (%) | 458 (90.2) | 479 (90.4) | .91 |
| Non-statin hypolipidemia agents, no (%) | 33 (6.5) | 35 (6.6) | .94 |

CPB, cardiopulmonary bypass; ICU, intense care unit; FBG, fasting blood glucose; ACEI, angiotensin converting enzyme inhibitors; ARB, angiotensin receptor blocker

**Table S2: Baseline Characteristics comparison by Study Completion**

| **Variables** | **Completed** | **Uncompleted** | ***P*-value** |
| --- | --- | --- | --- |
|  | **n = 1038** | **n = 28** |  |
| Female, no (%) | 210 (20.2) | 8 (28.6) | .28 |
| Age (years), mean (SD) | 60.9 (8.2) | 59.3 (9.6) | .46 |
| BMI (kg/m2), median [Q1, Q3] | 25.7 [23.9, 27.7] | 26.2 [23.7, 28.6] | .68 |
| SBP (mmHg), median [Q1, Q3] | 133 [122, 146] | 131 [123, 145] | .58 |
| Cigarette & alcohol, no (%) | 486 (46.8) | 7 (25.0) | .02 |
| Hypertension, no (%) | 723 (69.7) | 19 (67.9) | .84 |
| Hyperlipidemia, no (%) | 806 (77.6) | 23 (82.1) | .57 |
| Prior stroke, no (%) | 98 (9.4) | 0 | .17 |
| NYHA class Ⅲ or Ⅳ, no (%) | 266 (25.6) | 11 (39.3) | .10 |
| Triple-vessel disease, no (%) | 958 (92.3) | 26 (92.9) | >.999 |
| LM disease, no (%) | 270 (26.0) | 10 (35.7) | .25 |
| Preoperative medication |  |  |  |
| Beta blocker, no (%) | 923 (88.9) | 25 (89.3) | >.999 |
| ACEI/ARB, no (%) | 378 (36.4) | 9 (32.1) | .64 |
| Statin, no (%) | 966 (93.1) | 26 (26.1) | >.999 |
| Non-statin hypolipidemia agents, no (%) | 169 (16.3) | 4 (14.3) | .98 |
| Hypoglycemic intervention |  |  |  |
| Oral medication, no (%) | 541 (52.1) | 15 (53.6) | .88 |
| Insulin, no (%) | 414 (39.9) | 13 (46.4) | .49 |
| None, no (%) | 83 (8.0) | 0 | .16 |
| Diabetic comorbidity, no (%) | 36 (3.5) | 1 (3.6) | >.999 |
| Preoperative laboratory |  |  |  |
| HbA_1C_ (%), median [Q1, Q3] | 7.5 [6.8, 8.4] | 7.4 [6.7, 8.4] | .95 |
| HbA_1C_ ≤7.0%, no (%) | 367 (35.4) | 11 (39.3) | .67 |
| Creatine (mmol/L), median [Q1, Q3] | 83.0 [73.0, 94.3] | 80.2 [70.8, 94.1] | .67 |
| FBG (mmol/L), median [Q1, Q3] | 7.0 [5.8, 8.6] | 7.8 [6.1, 10.0] | .14 |
| LDL-C (mmol/L), median [Q1, Q3] | 2.0 [1.6, 2.5] | 2.3 [1.9, 2.8] | .03 |
| Current residence |  |  |  |
| City, no (%) | 676 (65.1) | 15 (53.6) | .21 |
| Rural area, no (%) | 362 (34.9) | 13 (46.4) |  |
| Enrollment center |  |  |  |
| Fuwai Hospital, no (%) | 971 (93.5) | 26 (89.3) | .61 |
| Qingdao Fuwai Hospital, no (%) | 67 (6.5) | 3 (10.7) |  |
| Health insurance |  |  |  |
| Urban basic health insurance, no (%) | 420 (40.5) | 10 (35.7) | .61 |
| Rural health insurance, no (%) | 254 (24.5) | 11 (7.0) | .07 |
| Other health insurance, no (%) | 364 (35.1) | 7 (25.0) | .27 |
| Education level |  |  |  |
| Below senior high school, no (%) | 470 (45.3) | 14 (50.0) | .62 |
| Senior high school or specialty, no (%) | 439 (42.3) | 11 (39.3) | .75 |
| Bachelor’s degree or above, no (%) | 129 (12.4) | 3 (10.7) | >.999 |

BMI, body mass index; SBP, systolic blood pressure; NYHA, New York Heart Association; LM, left main; ACEI, angiotensin converting enzyme inhibitors; ARB, angiotensin receptor blocker; HbA_1C_, glycosylated hemoglobin; FBG, fasting blood glucose; LDL-C, low-density lipoprotein cholesterol

**Table S3. baseline clinical and social characteristic of patients assigned to the control group**

| **Variables** | **Complete** | **Withdraw** | ***P*-value** |
| --- | --- | --- | --- |
|  | **n = 508** | **n = 24** |  |
| Female, no (%) | 103 (20.3) | 5 (20.8) | >.999 |
| Age (years), mean ± SD | 60.9 ± 8.2 | 63.8 ± 7.2 | .07 |
| BMI (kg/m2), median [Q1, Q3] | 25.8 [23.8, 27.7] | 25.8 [22.8, 28.2] | .41 |
| SBP (mmHg), median [Q1, Q3] | 134 [123, 148] | 131 [111, 142] | .13 |
| Cigarette & alcohol, no (%) | 232 (45.7) | 9 (37.5) | .43 |
| Hypertension, no (%) | 355 (69.9) | 18 (75.0) | .59 |
| Hyperlipidemia, no (%) | 396 (78.0) | 18 (75.0) | .73 |
| Prior stroke, no (%) | 53 (10.4) | 5 (20.8) | .21 |
| NYHA class Ⅲ or Ⅳ, no (%) | 133 (26.2) | 6 (25.0) | .90 |
| Triple-vessel disease, no (%) | 471 (92.7) | 21 (87.5) | .58 |
| LM disease, no (%) | 133 (26.2) | 5 (20.6) | .56 |
| Preoperative medication |  |  |  |
| Beta blocker, no (%) | 457 (90.0) | 24 (100.0) | .20 |
| ACEI/ARB, no (%) | 191 (37.6) | 7 (29.2) | .40 |
| Statin, no (%) | 470 (92.5) | 23 (95.8) | .84 |
| Non-statin hypolipidemia agents, no (%) | 89 (17.5) | 3 (12.5) | .72 |
| Hypoglycemic intervention |  |  |  |
| Oral medication, no (%) | 259 (51.0) | 9 (37.5) | .20 |
| Insulin, no (%) | 214 (42.1) | 13 (54.2) | .24 |
| None, no (%) | 35 (6.9) | 2 (8.3) | >.999 |
| Diabetic comorbidity, no (%) | 17 (3.3) | 1 (4.2) | .57 |
| Preoperative laboratory |  |  |  |
| HbA1C (%), median [Q1, Q3] | 7.5 [6.7, 8.5] | 7.7 [6.9, 8.3] | .75 |
| HbA1C < 7.0%, no (%) | 169 (33.3) |  |  |
| Creatine (mmol/L), median [Q1, Q3] | 84.0 [72.4, 95.5] | 83.8 [76.1, 106.2] | .34 |
| FBG (mmol/L), median [Q1, Q3] | 7.1 [5.7, 8.8] | 6.4 [5.6, 7.6] | .27 |
| LDL-C (mmol/L), median [Q1, Q3] | 2.0 [1.6, 2.5] | 1.9 [1.4, 2.4] | .46 |
| Current residence |  |  |  |
| City, no (%) | 340 (66.9) | 15 (62.5) | .65 |
| Rural area, no (%) | 168 (33.1) | 9 (37.5) |  |
| Health insurance |  |  |  |
| Urban basic health insurance, no (%) | 205 (40.4) | 6 (25.0) | .13 |
| Rural health insurance, no (%) | 123 (24.2) | 6 (25.0) | .93 |
| Self-pay, no (%) | 6 (1.2) | 1 (4.2) | .28 |
| Other health insurance, no (%) | 174 (34.3) | 11 (45.8) | .24 |
| Education level |  |  |  |
| Below senior high school, no (%) | 227 (44.7) | 11 (45.8) | .91 |
| Senior high school or specialty, no (%) | 216 (42.5) | 12 (50.0) | .47 |
| Bachelor degree, no (%) | 63 (12.4) | 1 (4.2) | .37 |
| Master degree or above, no (%) | 2 (0.4) | 0 (0.0) | >0.999 |

BMI, body mass index; SBP, systolic blood pressure; NYHA, New York Heart Association; LM, left main; ACEI, angiotensin converting enzyme inhibitors; ARB, angiotensin receptor blocker; HbA1C, glycosylated hemoglobin; FBG, fasting blood glucose; LDL-C, low-density lipoprotein cholesterol

**Table S4. Baseline Characteristics According to Final Analysis Status**

| **Variables** | **With HbA_1C_ results** | **Without HbA_1C_ results** | ***P*-value** |
| --- | --- | --- | --- |
|  | **n = 1000** | **n = 38** |  |
| Female, no (%) | 203 (20.3) | 7 (18.4) | .78 |
| Age (years), mean ± SD | 60.9 ± 8.2 | 61.7 ± 8.9 | .54 |
| BMI (kg/m2), median [Q1, Q3] | 25.7 [23.9, 27.7] | 25.7 [23.8, 28.1] | .67 |
| SBP (mmHg), median [Q1, Q3] | 133.0 [122.0, 146.0] | 135.0 [124.3, 152.3] | .42 |
| Cigarette & alcohol, no (%) | 464 (46.4) | 22 (57.9) | .16 |
| Hypertension, no (%) | 694 (69.4) | 29 (76.3) | .36 |
| Hyperlipidemia, no (%) | 776 (77.6) | 30 (78.9) | .85 |
| Prior stroke, no (%) | 95 (9.5) | 3 (7.9) | .96 |
| NYHA class Ⅲ or Ⅳ, no (%) | 259 (25.9) | 7 (18.4) | .30 |
| Triple-vessel disease, no (%) | 921 (92.1) | 37 (97.4) | .38 |
| LM disease, no (%) | 260 (26.0) | 10 (26.3) | .97 |
| Preoperative medication |  |  |  |
| Beta blocker, no (%) | 892 (89.2) | 31 (81.6) | .23 |
| ACEI/ARB, no (%) | 362 (36.2) | 16 (42.1) | .46 |
| Statin, no (%) | 929 (92.9) | 37 (97.4) | .46 |
| Non-statin hypolipidmia agents, no (%) | 164 (16.4) | 5 (13.2) | .60 |
| Hypoglycemic intervention |  |  |  |
| Oral medication, no (%) | 521 (52.1) | 20 (52.6) | .95 |
| Insulin, no (%) | 398 (39.8) | 16 (42.1) | .78 |
| None, no (%) | 81 (8.1) | 2 (5.3) | .74 |
| Diabetic comorbidity, no (%) | 34 (3.4) | 2 (5.3) | .38 |
| Preoperative laboratory |  |  |  |
| HbA1C (%), median [Q1, Q3] | 7.5 [6.7, 8.5] | 7.6 [6.9, 8.3] | .63 |
| HbA1C < 7.0%, no (%) | 322 (32.2) | 11 (28.9) | .67 |
| Creatine (mmol/L), median [Q1, Q3] | 83.0 [72.9, 94.0] | 92.1 [76.3, 106.0] | .04 |
| FBG (mmol/L), median [Q1, Q3] | 7.0 [5.8, 8.6] | 6.4 [5.5, 7.4] | .29 |
| LDL-C (mmol/L), median [Q1, Q3] | 2.0 [1.6, 2.5] | 2.0 [1.7, 2.7] | .06 |
| Current residence |  |  |  |
| City, no (%) | 652 (65.2) | 24 (63.2) | .80 |
| Rural area, no (%) | 348 (34.8) | 14 (36.8) |  |
| Health insurance |  |  |  |
| Urban basic health insurance, no (%) | 403 (40.3) | 17 (44.7) | .58 |
| Rural health insurance, no (%) | 244 (24.4) | 10 (26.3) | .79 |
| Self-pay, no (%) | 13 (1.3) | 0 | >.999 |
| Other health insurance, no (%) | 340 (34.0) | 11 (28.9) | .52 |
| Education level |  |  |  |
| Below senior high school, no (%) | 450 (45.0) | 20 (52.6) | .35 |
| Senior high school or specialty, no (%) | 427 (42.7) | 12 (31.6) | .17 |
| Bachelor degree, no (%) | 116 (11.6) | 6 (15.8) | .60 |
| Master degree or above, no (%) | 7 (0.7) | 0 | >.999 |

BMI, body mass index; SBP, systolic blood pressure; NYHA, New York Heart Association; LM, left main; ACEI, angiotensin converting enzyme inhibitors; ARB, angiotensin receptor blocker; HbA1C, glycosylated hemoglobin; FBG, fasting blood glucose; LDL-C, low-density lipoprotein cholesterol

**Table S5. ITT analysis of primary and secondary outcomes**

| **Variables** | **Control group** | | | **Intervention group** | | | **Adjusted mean difference in change** | **Adjusted *P*-value** |
| --- | --- | --- | --- | --- | --- | --- | --- | --- |
|  | **Baseline** | **Follow-up** | **Change** | **Baseline** | **Follow-up** | **Change** |  |  |
| HbA_1C_ | 7.7 (7.6- 7.8) | 7.2 (7.1 – 7.3) | -0.5 (-0.6 - -0.4) | 7.8 (7.6 – 7.9) | 7.1 (7.0 – 7.1) | -0.7 (-0.8 - -0.6) | -0.1 (-0.3 - -0) | .03 |
| HbA_1C_≤7.0% | 183 (36.0) | 262 (51.6) | / | 184 (34.7) | 301 (56.8) | / | 1.1 (1.0, 1.1) | .08 |
| SBP | 134 [123, 148] | 130 [120, 138] | -3 [-16, 3] | 133 [122, 145] | 130 [120, 137] | 0 [-14, 5.] | -0.2 (-1.7, 1.3） | .77 |
| FBG | 7.1 [5.7, 8.8] | 7.1 [6.2, 8.1] | 0.2 [-1.6, 1.4] | 6.9 [6.0, 8.4] | 6.9 [6.1, 7.9] | 0 [-1.6, 1.4] | -0.1 (-0.4, 0.1) | .26 |
| LDL-C | 2.0 [1.6, 2.5] | 2.2 [1.7, 2.7] | 0 [-0.2, 0.6] | 2.0 [1.6, 2.5] | 2.1 [1.8, 2.8] | 0 [-0.2, 0.6] | 0 (0, 0.1) | .58 |

**Table S6. Exploratory outcomes**

| **Variables** | **Control group** | **Intervention group** | ***P*-value** |
| --- | --- | --- | --- |
|  |  |  |  |
| **Exploratory outcomes** |  |  |  |
| ΔHbA1C＜0.5% n, (%) | 225 (46.3) | 282 (54.9) | .03^a^ |
| Self-report medical adherence |  |  |  |
| Hardly forget | 411 (93.2) | 449 (96.1) | .047 |
| Occasionally forget | 30 (6.8) | 16 (3.4) |  |
| EQ-5D score | 0.957 ± 0.09 | 0.962 ± 0.08 | .69 |
| EQ-VAS | 86.0 [80.0, 95.0] | 88.0 [80.0, 95.0] | .52 |
| MACCE | 10 (2.0) | 12 (2.3) | .74^b^ |

EQ-5D: EuroQol five-dimensional questionnaire, EQ-VAS: EuroQol visual analogue scale, MACCE: major adverse cardiac cerebrovascular events.

a Tested by Chi-squared test.

b Tested by log-rank.

**Table S7. Factors related to more frequent use of APP**

| **Variables** | **Univariate regression** | | | **Multivariate regression** | | | |
| --- | --- | --- | --- | --- | --- | --- | --- |
|  | **OR** | **95% CI** | ***P*-value** | **OR** | **95% CI** | ***P*-value** | **VIF** |
| Female | 1.79 | 1.05 - 3.08 | .03 | 1.10 | 0.59 - 2.03 | .77 | 1.266 |
| Urban area residence | 0.48 | 0.29 - 0.77 | .003 | 0.51 | 0.31 - 0.83 | .007 | 1.032 |
| Cigarette & alcohol | 0.45 | 0.27 - 0.75 | .002 | 0.50 | 0.28 - 0.88 | .02 | 1.262 |
| ACEI/ARB | 1.56 | 0.96 - 2.54 | .07 | 1.61 | 0.97 - 2.66 | .06 | 1.013 |
| Baseline HbA_1C_≤7.0% | 0.51 | 0.29 - 0.89 | .02 | 0.54 | 0.31 - 0.96 | .04 | 1.022 |

ACEI, angiotensin converting enzyme inhibitors; ARB, angiotensin receptor blocker; HbA_1C_, glycosylated hemoglobin;

**Table S7.** **Subgroup analysis based on APP usage intensity**

| **Subgroup** | **No. patients** | **APP open count** | ***P*-value** | **Active user** | ***P*-value** |
| --- | --- | --- | --- | --- | --- |
| *Age* |  |  | .11 |  | >.999 |
| < 60 yrs | 229 | 5.0 [3.0,9.0] |  | 34(14.8%) |  |
| ≥ 60 yrs | 301 | 6.0 [3.0,10.0] |  | 45(15.0%) |  |
| *Sex* |  |  | .06 |  | .047 |
| Male | 107 | 5.0 [3.0,9.0] |  | 45(15.0%) |  |
| Female | 423 | 6.0 [3.0,11.0] |  | 23(21.5%) |  |
| *Area* |  |  | .11 |  | .003 |
| Urban | 336 | 5.0 [3.0,9.0] |  | 38(11.3%) |  |
| Rural | 149 | 6.0 [3.0,11.0] |  | 41(21.1%) |  |
| *Educational level* | |  | .33 |  | .26 |
| ≤12 yrs | 446 | 6.0 [3.0,10.0] |  | 73(15.7%) |  |
| > 12 yrs | 64 | 5.0[3.0,8.0] |  | 6(9.4%) |  |
| *Tertiles baseline HbA_1C_* | |  | .002 |  | .050 |
| Q1 (0, 7.0] | 184 | 5.0 [2.0,7.2] |  | 18(9.8%) |  |
| Q2 (7.0, 8.0) | 163 | 6.0 [3.0,10.0] |  | 30(18.4%) |  |
| Q3 [8.0, max] | 183 | 6.0 [3.0,10.0] |  | 31(16.9%) |  |

APP opens are presented as median [75th percentile]. An active user is defined as one who opened the app 12 or more times during the study period.

**Table S8.** **Comparison of primary outcome between active and non-active users across different subgroups**

| **Subgroup** | **ΔHbA_1C_** | | **Mean difference in change** | ***P*-value** |
| --- | --- | --- | --- | --- |
|  | **Active user** | **Non-active user** |  |  |
| Age |  |  |  |  |
| < 60 yrs | -1.30±1.20 | -0.63±1.58 | 0.6 (0.2, 1.0) | .006 |
| ≥ 60 yrs | -1.44±1.18 | -0.57±1.54 | 0.9 (0.5, 1.3) | <.001 |
| Sex |  |  |  |  |
| Male | -1.33±1.33 | -0.52±1.40 | 0.7 (0.4, 1.0) | <.001 |
| Female | -1.50±0.67 | -0.93±2.07 | 0.8 (0.3, 1.2) | .002 |
| Area |  |  |  |  |
| Urban | -1.24±0.91 | -0.54±1.56 | 0.7 (0.4, 1.0) | <.001 |
| Rural | -1.51±1.41 | -0.70±1.54 | 0.8 (0.3, 1.2) | .003 |
| Educational level | |  |  |  |
| ≤12 yrs | -1.42±1.22 | -0.58±1.60 | 0.8 (0.6, 1.1) | <.001 |
| > 12 yrs | -0.87±0.32 | -0.70±1.21 | 0.3 (-0.8, 1.0) | .50 |
| Tertiles baseline HbA1C | |  |  |  |
| Q1 (0, 7.0] | -0.50±0.69 | 0.24±0.79 | 0.7 (0.4, 1.1) | <.001 |
| Q2 (7.0, 8.0) | -1.17±0.42 | -0.32±1.10 | 0.7 (0.4, 1.0) | <.001 |
| Q3 [8.0, max] | -2.07±1.45 | -1.76±1.81 | 0.2 (-0.3, 0.7) | 0.38 |

Changes in HbA1c are presented as mean ± standard deviation. The mean difference in change is expressed as the mean (95% confidence interval).

**Table S9.** **Mean, standard deviation, and standard error of continuous variables for primary and secondary outcomes**

| **Variables** | | | **HbA_1C_** | **SBP** | **FBG** | **LDL** |
| --- | --- | --- | --- | --- | --- | --- |
| **Control group** | **Baseline** | **Mean** | 7.7 | 135.5 | 7.5 | 2.1 |
|  |  | **SD** | 1.4 | 19.0 | 2.6 | 0.9 |
|  |  | **SE** | 0.1 | 0.9 | 0.1 | 0.0 |
|  | **Follow-up** | **Mean** | 7.2 | 128.9 | 7.4 | 2.3 |
|  |  | **SD** | 1.0 | 12.0 | 2.0 | 0.7 |
|  |  | **SE** | 0.0 | 0.6 | 0.1 | 0.0 |
|  | **Change** | **Mean** | -0.5 | -6.6 | -0.2 | 0.2 |
|  |  | **SD** | 1.4 | 20.4 | 2.8 | 1.0 |
|  |  | **SE** | 0.1 | 1.0 | 0.1 | 0.0 |
| **Intervention group** | **Baseline** | **Mean** | 7.8 | 133.5 | 7.4 | 2.1 |
|  |  | **SD** | 1.5 | 17.1 | 2.4 | 0.8 |
|  |  | **SE** | 0.1 | 0.8 | 0.1 | 0.0 |
|  | **Follow-up** | **Mean** | 7.0 | 128.3 | 7.2 | 2.0 |
|  |  | **SD** | 1.1 | 10.9 | 1.9 | 0.7 |
|  |  | **SE** | 0.0 | 0.5 | 0.1 | 0.0 |
|  | **Change** | **Mean** | -0.7 | -0.5 | -0.2 | 0.2 |
|  |  | **SD** | 1.5 | 19.1 | 2.8 | 0.9 |
|  |  | **SE** | 0.1 | 0.9 | 0.1 | 0.0 |
